# Infected splenic artery pseudoaneurysm within a complex splenic cystic lesion: CT angiography pitfalls and surgical correlation

**DOI:** 10.1016/j.radcr.2026.08.003

**Published:** 2026-09-01

**Authors:** Rihab Amara, Nawal Chyabri, Nadia Mouna, Tarik Deflaoui, Mouataz Aabalou, Hamid Ziani, Siham Nasri, Imane Skiker

**Affiliations:** a Department of Radiology, Mohammed VI University Hospital, Oujda, Morocco; b Department of General Surgery, Mohammed VI University Hospital, Oujda, Morocco

**Keywords:** Splenic lesion, Pseudoaneurysm, CT angiography, Splenectomy, Infected cyst, Vascular complication

## Abstract

Complex cystic splenic lesions can pose considerable diagnostic difficulty because infectious, hemorrhagic, and neoplastic processes frequently show overlapping imaging features, and splenic artery pseudoaneurysm is a rare but potentially fatal vascular complication requiring prompt recognition. We report the case of an 85-year-old male who presented with a persistent systemic inflammatory syndrome; contrast-enhanced multiphasic CT and CT angiography with 3D reconstruction demonstrated a large complex cystic splenic lesion harboring a splenic artery pseudoaneurysm, and, given the high risk of rupture, the patient underwent splenectomy with vascular control, with surgical and histopathological findings confirming an infected splenic cystic lesion complicated by splenic artery pseudoaneurysm without evidence of malignancy and microbiological culture of the infected collection yielding *Lancefieldella rimae*. This case illustrates the value of arterial-phase CT and CT angiography in differentiating pseudoaneurysmal enhancement from hemorrhagic or inflammatory cystic splenic lesions, and underscores that early recognition is critical to guide surgical planning and to prevent catastrophic hemorrhagic rupture.

## Introduction

Splenic cystic lesions represent a heterogeneous group of rare entities including epithelial cysts, pseudocysts, abscesses, and neoplastic lesions. Their diagnosis is often challenging due to overlapping imaging features.

Splenic artery pseudoaneurysm is a rare but severe vascular abnormality most associated with pancreatitis, trauma, or infection. Infection-related pseudoaneurysm formation is particularly uncommon but associated with high rupture risk and mortality [1].

CT angiography is the gold standard for diagnosis and surgical planning [1,2].

We report a rare case of a cystic splenic lesion complicated by an infected splenic artery pseudoaneurysm.

## Case presentation

### Clinical data

An 85-year-old male was admitted for persistent systemic inflammatory syndrome of unknown origin. His medical history included type 2 diabetes mellitus treated with insulin and oral antidiabetic agents.

### Laboratory findings

Laboratory investigations at admission showed mild anemia (hemoglobin 11 g/dL) with a leukocytosis of 13 × 10⁹/L characterized by neutrophilia (75%) and relative lymphopenia (15%), a normal platelet count (157 × 10⁹/L), and a markedly elevated C-reactive protein (60 mg/L); the erythrocyte sedimentation rate was not performed. Liver function tests, renal function, tumor markers, blood cultures, and hydatid serology were within normal limits or negative, as detailed in Table 1. Microbiological culture of the intraoperative specimen obtained from the infected splenic collection subsequently yielded *Lancefieldella rimae*, an anaerobic Gram-positive coccus, supporting the infectious nature of the lesion (Table 1).

**Table 1:** Summary of laboratory findings at admission.

Parameter	Patient value	Reference range
Hemoglobin	11 g/dL	13.0-17.0 g/dL
White blood cell count	13 × 10⁹/L	4.0-11.0 × 10⁹/L
Neutrophils	75%	40%-70%
Lymphocytes	15%	20%-40%
Platelet count	157 × 10⁹/L	150-450 × 10⁹/L
C-reactive protein (CRP)	60 mg/L	<5 mg/L
Erythrocyte sedimentation rate (ESR)	Not performed	<20 mm/h
AST/ALT	10 U/L/9 U/L	AST 10-40 U/L; ALT 7-56 U/L
Total bilirubin	0.8 mg/dL	0.2-1.2 mg/dL
Serum creatinine/eGFR	0.8 mg/dL/eGFR > 60 mL/min	0.6-1.2 mg/dL; eGFR > 60 mL/min
Tumor markers (CEA, CA 19-9)	Within normal limits	—
Blood cultures	Negative	—
Hydatid serology	Negative	—
Intraoperative specimen culture	*Lancefieldella rimae* isolated	—

### Imaging findings

Contrast-enhanced multiphasic CT demonstrated a large complex cystic splenic lesion with heterogeneous internal content and marked surrounding inflammatory changes.

Arterial-phase imaging demonstrated a focal intralesional hyperattenuating component showing attenuation values comparable to the splenic artery, highly suggestive of active pseudoaneurysmal enhancement rather than hemorrhagic debris. The intralesional vascular focus measured approximately 165 HU during arterial-phase imaging, similar to attenuation values measured within the splenic artery. Persistent enhancement on portal venous phase images further supported vascular communication. On multiplanar reformatted images the lesion measured approximately 4.0 cm in craniocaudal extent, with a maximal diameter of 2.1 cm. No frank active contrast extravasation (ie, progressive extraluminal pooling of contrast into the adjacent cystic cavity on delayed-phase images) was identified; instead, the vascular focus remained a well-circumscribed, rounded structure with attenuation paralleling that of the splenic artery throughout all contrast-enhanced phases, favoring a contained pseudoaneurysm rather than active hemorrhage. CT angiography demonstrated that the pseudoaneurysm arose from the main trunk of the splenic artery, at its distal segment proximal to the splenic hilum, rather than from a segmental or distal branch vessel, and was embedded within the wall of the cystic cavity.

**Fig. 1:**
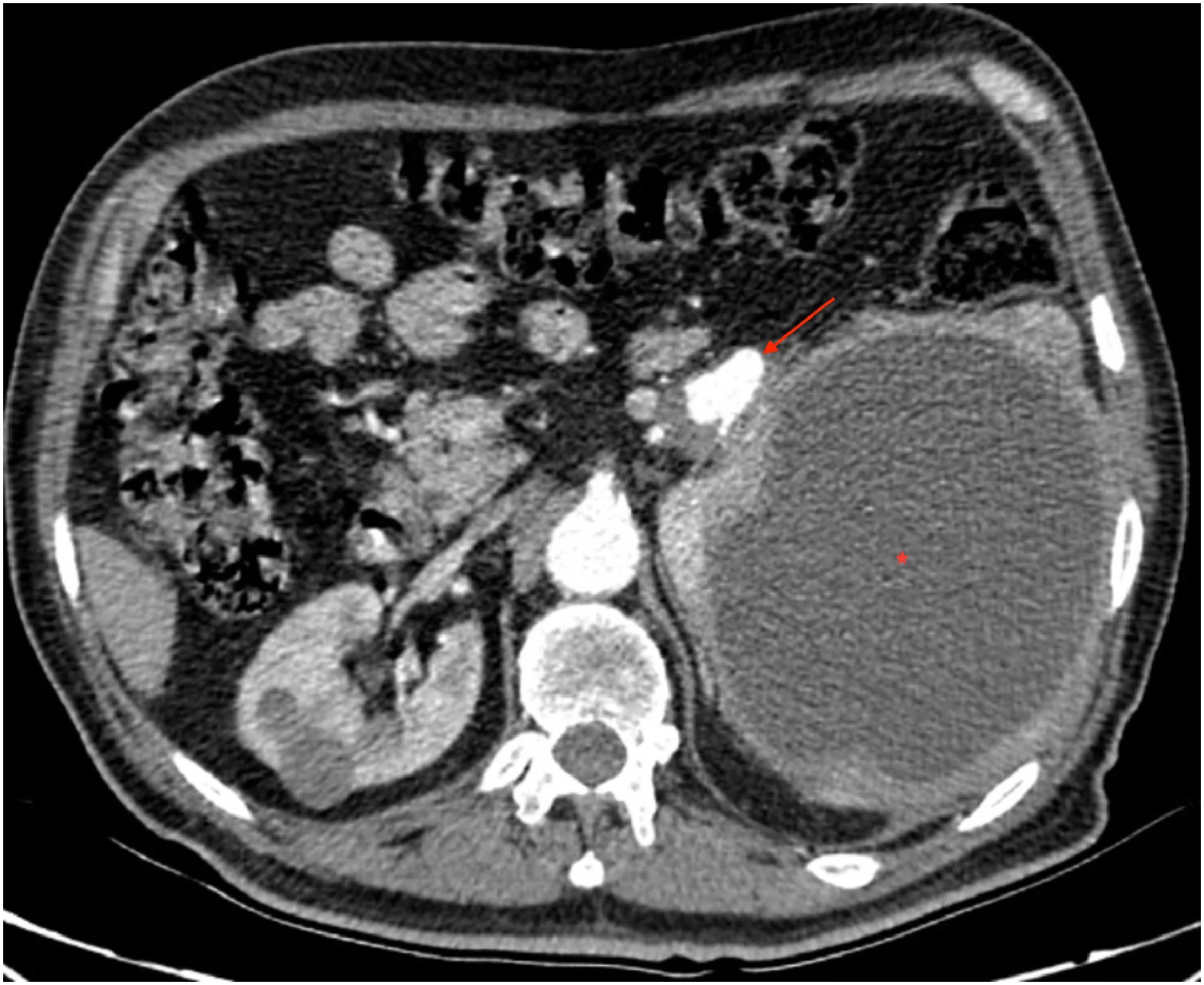
Axial contrast-enhanced CT image, arterial phase, demonstrating a large cystic splenic lesion (asterisk) with associated intralesional inflammatory changes consistent with abscess formation. Arrow indicates a focal vascular structure within the lesion suggestive of a splenic artery pseudoaneurysm.

**Fig. 2:**
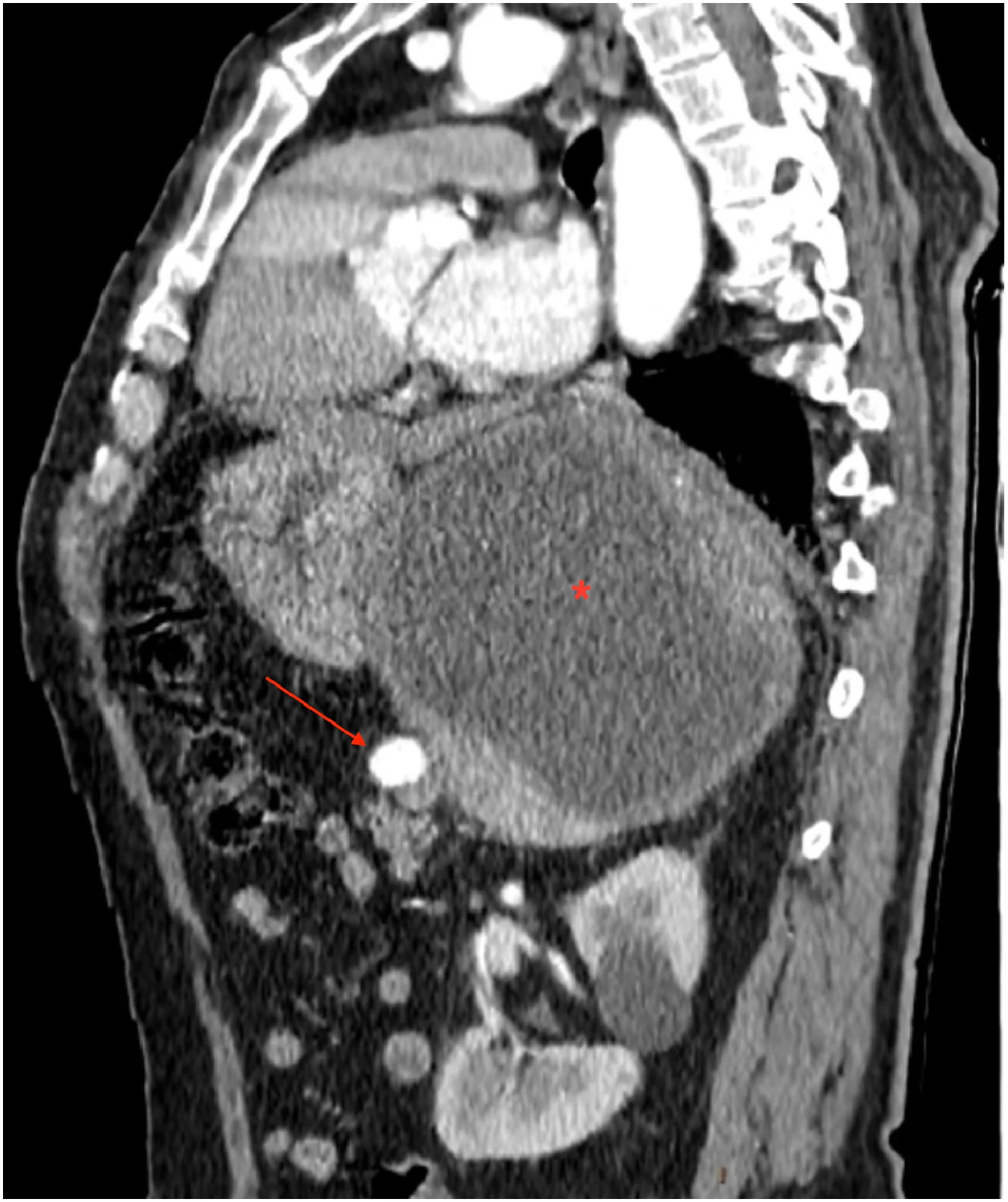
Sagittal contrast-enhanced CT reconstruction, arterial phase, showing the extent of the cystic splenic lesion (asterisk) with surrounding inflammatory changes. Arrow indicates vascular enhancement within the lesion, compatible with a pseudoaneurysm of the splenic artery.

**Fig. 3:**
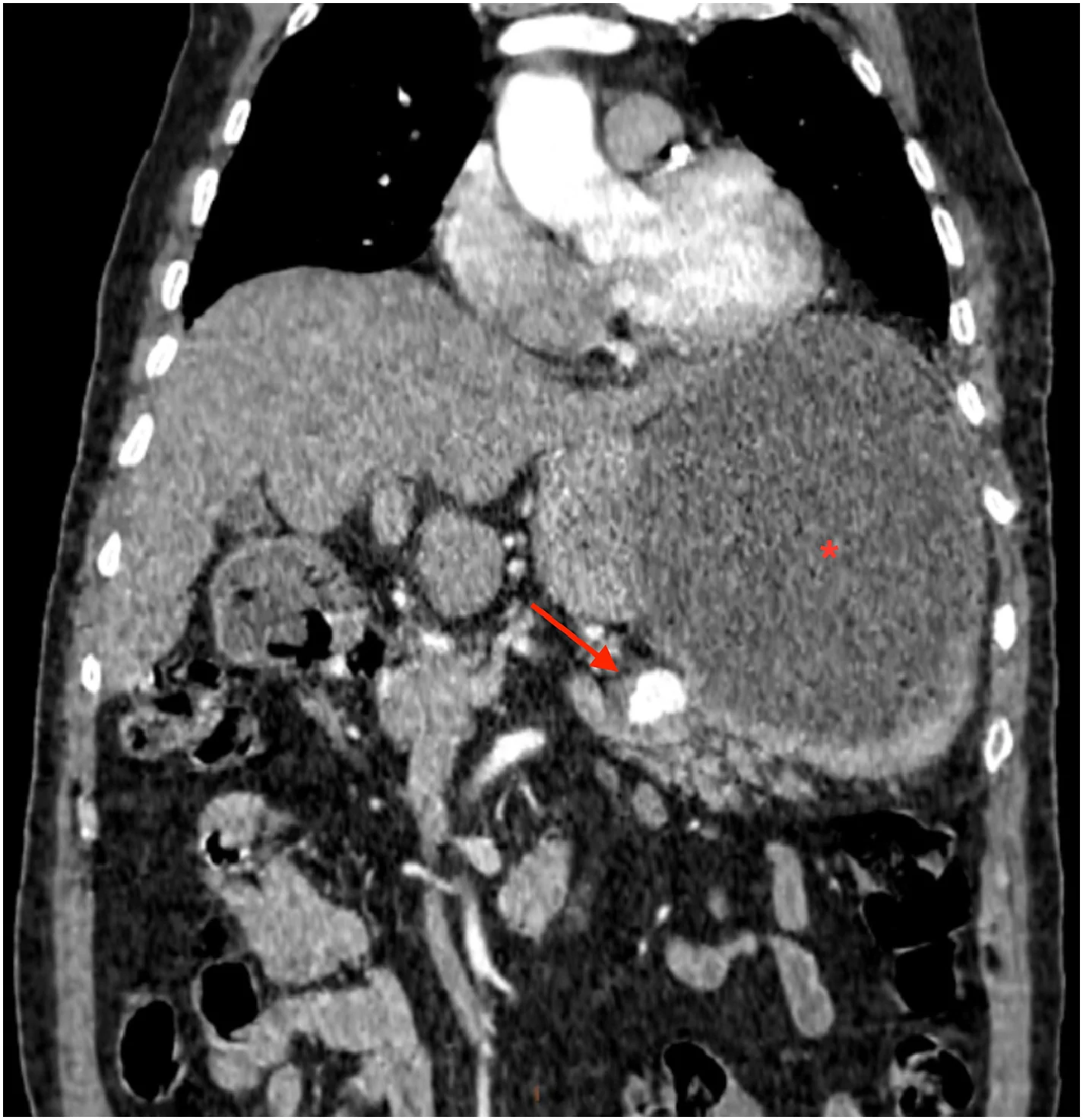
Coronal contrast-enhanced CT reconstruction, portal venous phase, demonstrating the relationship between the large cystic splenic lesion (asterisk) and adjacent abdominal structures. Arrow indicates the inflammatory/vascular component, including the suspected pseudoaneurysm.

**Fig. 4:**
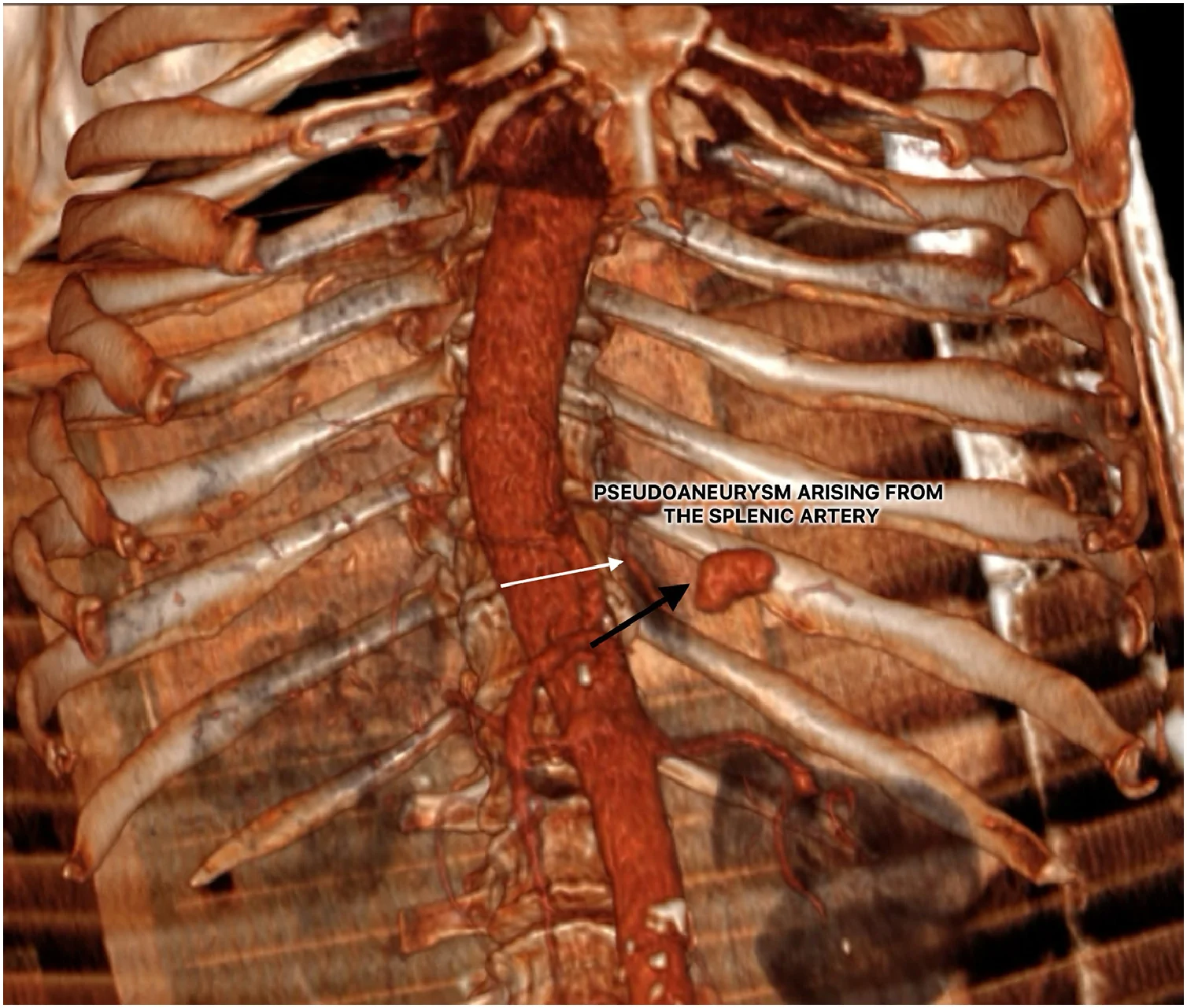
CT angiography with 3D volume-rendered reconstruction. White arrow indicates the splenic artery, and black arrow indicates the neck of the pseudoaneurysm.

CT angiography with multiplanar (Video. 1) and 3D volume-rendered reconstructions (Video. 2) demonstrated a splenic artery pseudoaneurysm embedded within the inflammatory splenic cavity and clearly delineated its anatomical relationship with adjacent vascular structures; as this reconstruction depicts only the contrast-opacified vascular lumen, the nonenhancing cystic component of the lesion is instead best appreciated on the cross-sectional images (Fig. 1, Fig. 2, Fig. 3, Fig. 4).

The combination of arterial enhancement, persistence on delayed phases, and vascular continuity favored the diagnosis of splenic artery pseudoaneurysm complicating an infected cystic splenic lesion.

### Differential diagnosis

The differential diagnosis included splenic abscess complicated by pseudoaneurysm, infected splenic cyst (post-traumatic or congenital), hydatid cyst (rendered unlikely by negative serology and the absence of daughter cysts or a calcified wall), cystic splenic neoplasm, and a hemorrhagic splenic lesion with underlying vascular injury.

## Management and outcome

Given the high risk of rupture, surgical management was indicated.

**Fig. 5:**
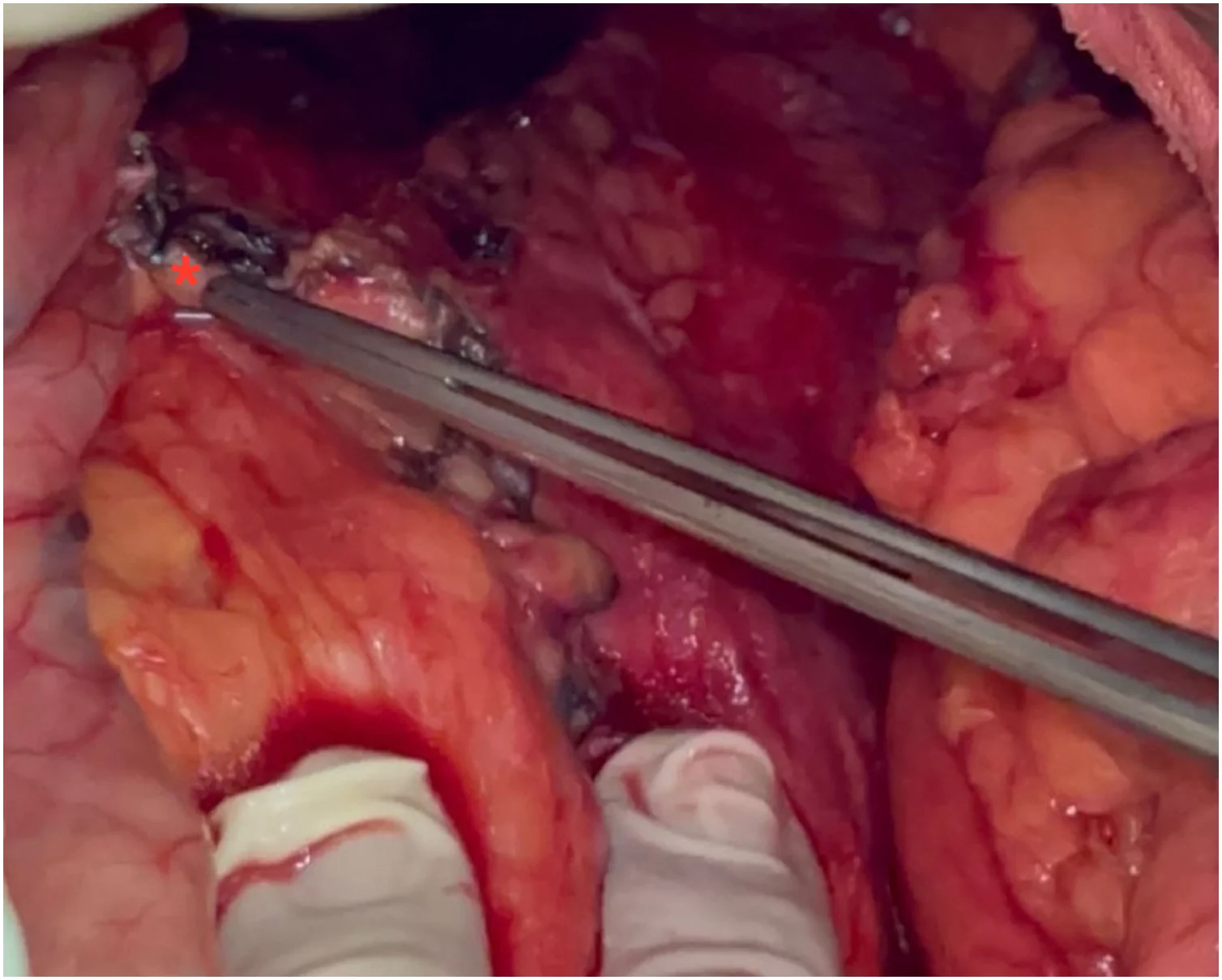
Intraoperative photograph demonstrating the vascular abnormality (asterisk) consistent with a splenic artery pseudoaneurysm.

**Fig. 6:**
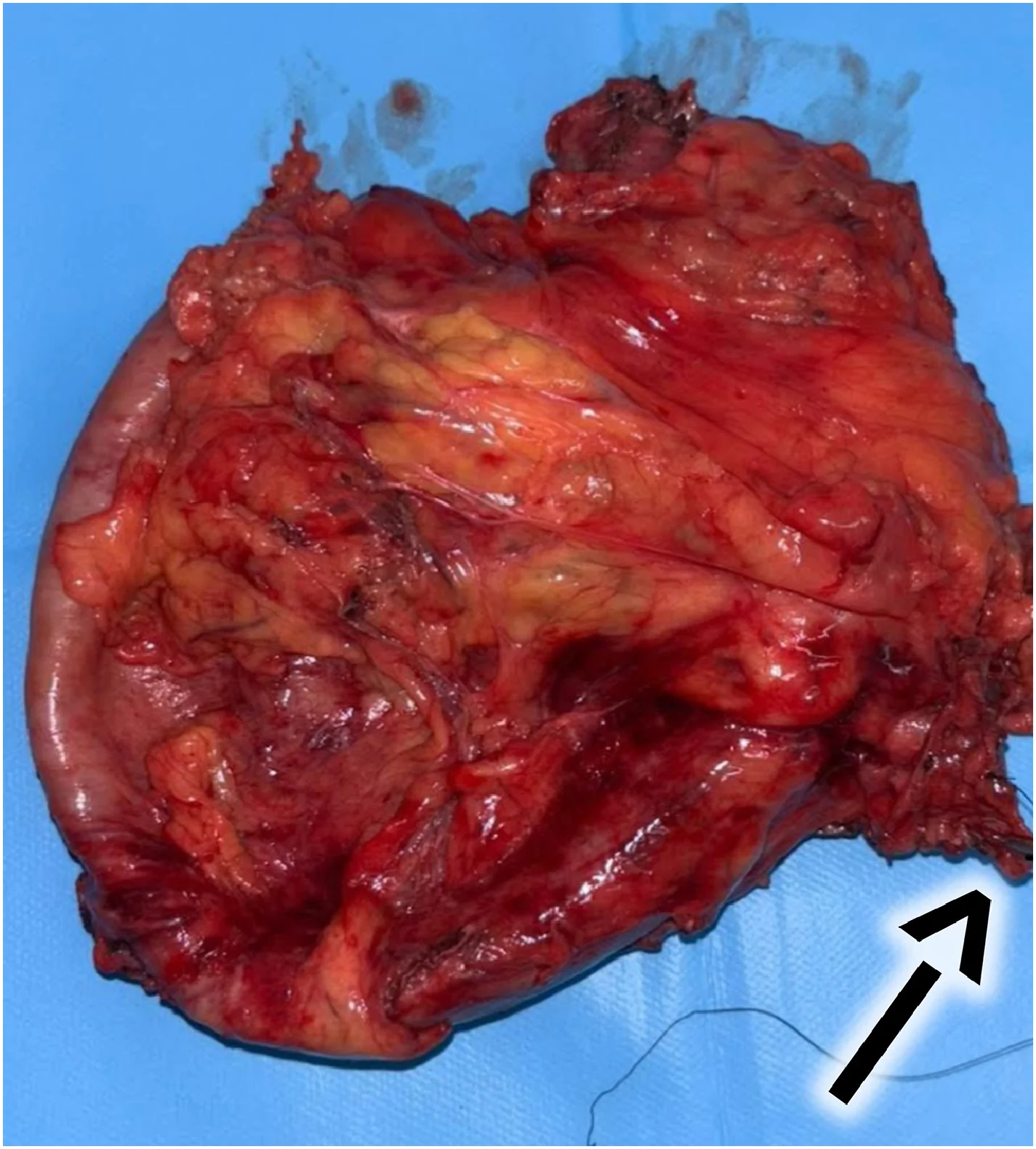
Resected specimen showing the pseudoaneurysmal lesion of the splenic artery, indicated by arrows.

**Fig. 7:**
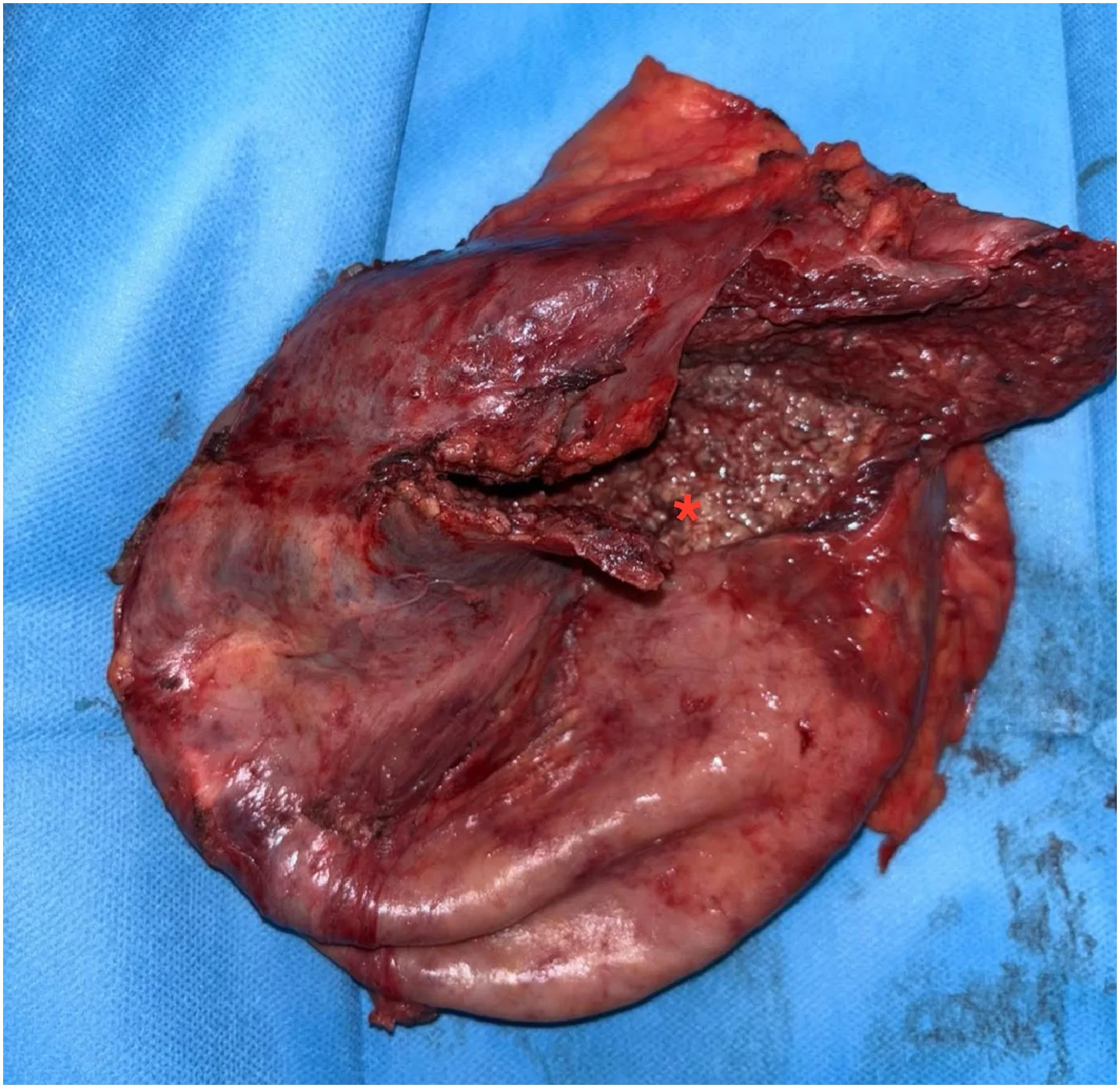
Gross specimen demonstrating the residual cavity of the splenic abscess (asterisk) after surgical resection.

The patient underwent total splenectomy with control of the splenic artery pseudoaneurysm, together with peritoneal lavage and drainage (Fig. 5, Fig. 6, Fig. 7).

### Intraoperative findings

Intraoperative exploration (Video. 3) confirmed a large cystic splenic lesion with an infected cavity containing necrotic material and a vascular lesion corresponding to the pseudoaneurysm identified on preoperative imaging, without evidence of malignancy.

### Pathology

Histopathological examination confirmed an infected cystic splenic lesion with inflammatory and vascular wall changes consistent with pseudoaneurysm formation, without malignant cells. Microbiological analysis of the intraoperative specimens yielded *L rimae*, an anaerobic Gram-positive coccus, supporting the infectious nature of the splenic lesion and its associated vascular complication.

## Discussion

Complex cystic splenic lesions encompass a broad spectrum of congenital, traumatic, infectious, and neoplastic entities with frequently overlapping imaging features [1,2]. Superinfection or intralesional hemorrhage may further complicate radiological characterization.

Splenic artery pseudoaneurysm represents an uncommon but potentially life-threatening vascular complication resulting from focal arterial wall disruption. Most cases are associated with pancreatitis or trauma, whereas infection-related pseudoaneurysms remain exceptionally rare [3,4]. In the present case, microbiological isolation of *L rimae* supported an infectious mechanism contributing to arterial wall weakening and pseudoaneurysm formation.

The main diagnostic challenge was differentiating pseudoaneurysmal enhancement from hemorrhagic or inflammatory intralesional content. Arterial-phase CT played a decisive role by demonstrating focal enhancement with attenuation characteristics identical to the splenic artery and persistence on delayed images. CT angiography with multiplanar and 3D reconstructions further improved visualization of vascular continuity and spatial relationships within the inflammatory cavity [5].

Differential diagnoses included splenic abscess, hydatid disease, hemorrhagic cystic lesion, and cystic neoplasm. Although splenic abscess commonly presents as a hypodense lesion with peripheral inflammatory enhancement, the presence of focal arterial enhancement should raise suspicion for associated vascular erosion or pseudoaneurysm formation [6]. Hydatid disease was considered less likely because no daughter cysts or calcified walls were identified and serology was negative [7].

Recognition of infected splenic artery pseudoaneurysm is essential because rupture risk is unpredictable and associated with high mortality [8]. While endovascular embolization may be considered in selected stable patients, splenectomy remains the preferred treatment in cases with extensive infection, tissue destruction, or large inflammatory cavities [9,10].

This case emphasizes the importance of integrating multiphasic CT findings, angiographic reconstructions, and clinical inflammatory context when evaluating complex splenic cystic lesions [11].

Reported cases of infected (mycotic) splenic artery pseudoaneurysm remain scarce and have most often been described in association with infective endocarditis or a contiguous splenic abscess, with splenectomy and vascular control generally required because of the substantial risk of rupture [12]. A comparable mechanism, whereby a localized infective or abscess-forming process seeds an adjacent visceral artery and produces a mycotic aneurysm, has also been reported for the celiac and splenic arteries in the setting of a perinephric abscess [13]. These reports, together with the present case, suggest that any localized infective or abscess-forming process adjacent to the splenic hilum should raise suspicion for vascular wall involvement, warranting dedicated arterial-phase and angiographic evaluation even when the clinical picture is dominated by nonspecific inflammatory symptoms.

## Conclusion

Infected splenic artery pseudoaneurysm complicating a cystic splenic lesion is a rare but potentially fatal condition. Multiphasic CT and CT angiography are essential for identifying pseudoaneurysmal enhancement and differentiating vascular complications from hemorrhagic or inflammatory cystic lesions. Early diagnosis is critical for appropriate surgical planning and prevention of catastrophic rupture. Splenic cystic lesions may be complicated by a life-threatening vascular pseudoaneurysm, and infection—although rare—is an important cause of splenic artery pseudoaneurysm formation. Because imaging may mimic an abscess or a hemorrhagic cystic tumor, CT angiography remains essential for preoperative vascular characterization; early recognition prevents fatal splenic rupture and guides appropriate surgical management.

## Patient consent

The authors confirm that written informed consent was obtained from the patient for publication of this case report and any accompanying images, in accordance with the ethical standards of Radiology Case Reports (Elsevier).

## Appendix Supplementary materials

Video 1. Axial CT cine loop, arterial and portal venous phases, demonstrating a large cystic splenic lesion with internal inflammatory changes consistent with abscess formation and an associated vascular component suggestive of splenic artery pseudoaneurysm. Image, video 1 dummy alt text

Video 2. CT angiography 3D reconstruction video showing a splenic artery pseudoaneurysm arising in close relationship with a complex cystic splenic lesion. Image, video 2 dummy alt text

Video 3. Intraoperative video demonstrating a pseudoaneurysm of the splenic artery within the operative field. The vascular lesion is visualized adjacent to the splenic cystic lesion, confirming the preoperative CT angiography findings and illustrating its intimate relationship with the surrounding inflammatory tissue. Image, video 3 dummy alt text
